# Diagnosis of Cervical Distractive-Flexion Injury Based on Minor Facet Fracture and Subtle Widening of the Facet Joint Space: A Case Report

**DOI:** 10.7759/cureus.91144

**Published:** 2025-08-27

**Authors:** Akira Itoi, Hidetoshi Nojiri, Ryosuke Takahashi, Arihisa Shimura, Tomoya Kojou, Yuya Higashiura, Takahiro Ushimaki, Atsuhiko Mogami

**Affiliations:** 1 Department of Orthopaedic Surgery, Juntendo University Shizuoka Hospital, Izunokuni, JPN; 2 Department of Orthopaedic Surgery, Faculty of Medicine, Juntendo University, Bunkyo, JPN; 3 Department of Spine Surgery, Naruo Orthopaedic Surgeon Hospital, Kumamoto, JPN

**Keywords:** bone/diagnostic imaging, cervical vertebrae/injuries, distractive‑flexion injury, hidden fracture, spinal injuries

## Abstract

Cervical distractive-flexion (DF) injuries are occasionally overlooked on initial evaluation, leading to delayed diagnosis at the stage of dislocation or late-onset neurological symptoms. Although detailed evaluation of osseous injury has become possible with the widespread use of CT, diagnosis remains challenging when DF injuries are spontaneously reduced in the supine position during imaging. According to the Allen-Ferguson classification, minor fractures of the superior articular process are indicative of DF injuries; however, few reports mention the evaluation of such findings in cases of delayed dislocation. This report presents a case of DF injury (Stage 1) diagnosed by recognizing a minor fracture of the articular process and subtle widening of the contralateral facet joint, leading to appropriate imaging evaluation and timely diagnosis.

## Introduction

Cervical spine dislocations can spontaneously reduce, particularly in distractive-flexion (DF) injuries as classified by Allen et al. [1]. When such reductions occur before imaging, the injury may be missed or misdiagnosed, potentially leading to delayed dislocation and late-onset neurological symptoms [1,2]. Early and accurate detection of these injuries is therefore critical to ensure optimal clinical outcomes.

Advances in multiplanar reconstruction computed tomography (CT) have improved the detection of cervical osseous injuries. However, diagnosing DF injuries remains challenging when spontaneous reduction occurs, especially in patients imaged in the supine position [2,3]. In such cases, subtle radiographic findings may be the only indicators of underlying instability.

Although several cases of delayed dislocation have been reported [2,4-6], few studies have specifically examined the diagnostic value of minor facet fractures or subtle joint abnormalities. Notably, findings such as fractures of the facet tip or minimal widening of the facet joint space are often overlooked and are not included in current cervical spine injury classification systems [7].

This diagnostic challenge is particularly relevant in elderly trauma patients, in whom pre-existing degenerative changes may obscure subtle injury patterns. Furthermore, delayed or missed diagnoses of cervical instability have been repeatedly reported in the literature, sometimes leading to catastrophic neurological outcomes [1]. Recognizing even minor abnormalities - such as a facet tip fracture or slight contralateral facet widening - can therefore provide essential diagnostic clues. These points emphasize the importance of maintaining a high index of suspicion when evaluating apparently minor cervical trauma.

## Case presentation

**Figure 1:**
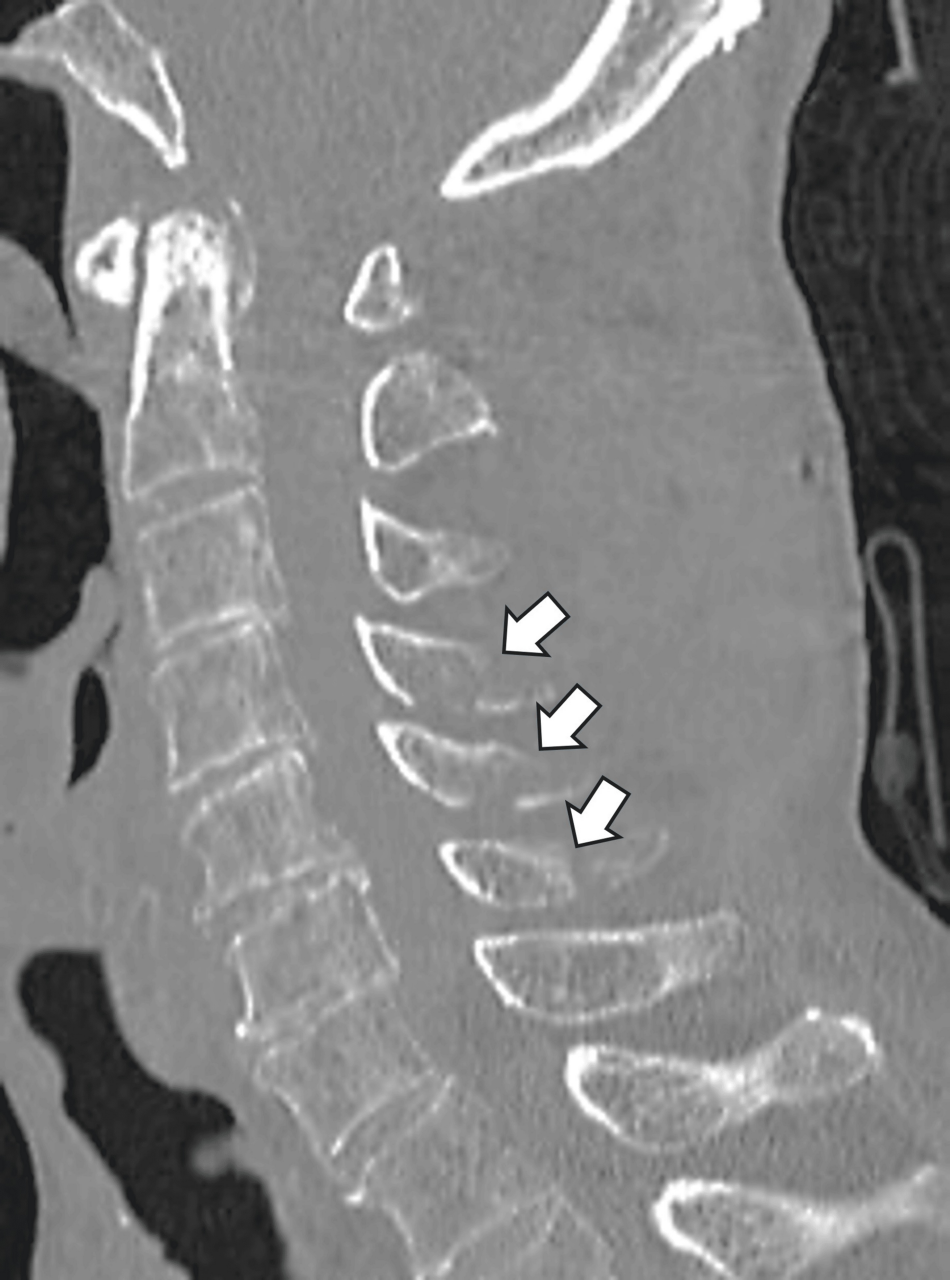
Sagittal reconstructed computed tomography (CT) image. Arrows point to tip fractures involving the superior and inferior articular processes at C4-C6.

**Figure 2:**
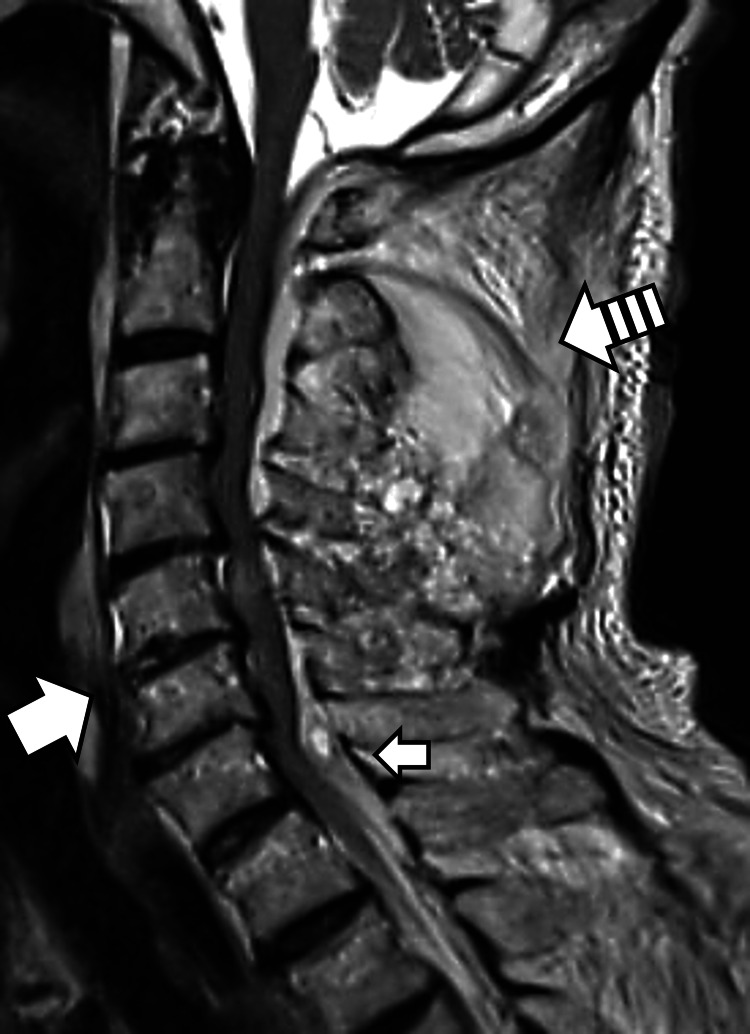
Sagittal T2-weighted magnetic resonance image (MRI). The thick arrow indicates anterior cervical hematoma. The thin arrow highlights the epidural hematoma. The striped arrow shows a soft tissue hematoma in the upper cervical spine.

**Figure 3:**
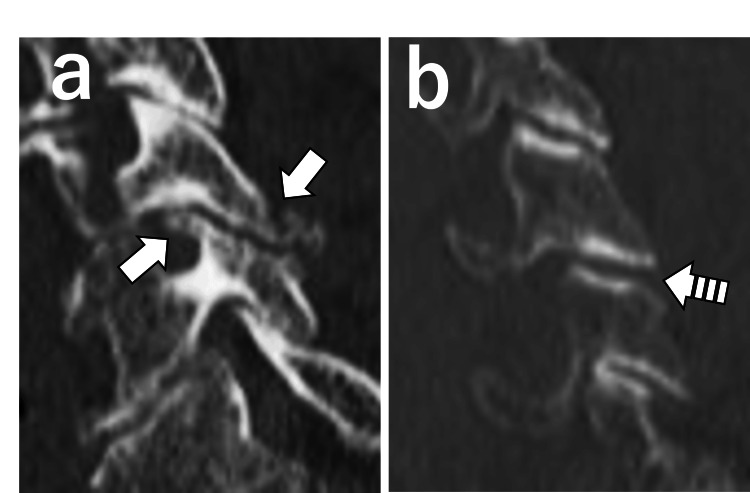
Parasagittal CT images. (a) Right-sided parasagittal image showing fractures of the superior and inferior articular processes (arrows). (b) Contralateral parasagittal image showing facet joint widening (striped arrow).

**Figure 4:**
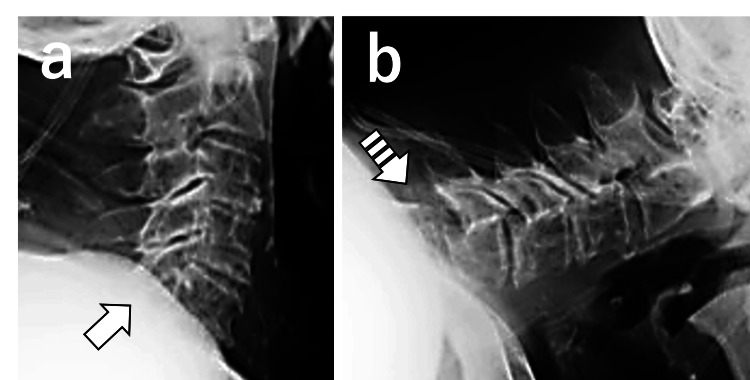
Dynamic lateral cervical spine radiographs taken with a portable X-ray machine. (a) Extension view in which the C5/6 alignment appears reduced. The facet joints are partially obscured by the shoulders. (b) Flexion view showing anterior subluxation at the C5/6 level (striped arrow), consistent with instability.

A 92-year-old man was transported to our hospital after a rear-end motor vehicle collision, complaining of posterior neck pain. He was initially diagnosed with a spinous process fracture based on CT (Figure 1), and magnetic resonance imaging (MRI) revealed an anterior cervical hematoma, epidural hematoma, and soft tissue hematoma in the upper cervical spine (Figure 2). However, re-evaluation of the parasagittal CT reconstruction by a spine surgeon revealed fractures of the tips of the superior and inferior articular processes at the C5/6 level (Figure 3a), along with contralateral facet joint widening (Figure 3b). These findings raised suspicion of underlying DF injury. Initial cervical flexion-extension testing was performed under the supervision of an orthopaedic surgeon, using portable dynamic radiography. During flexion, he experienced radiating pain in the upper limb, and radiography demonstrated anterior subluxation at the level of the suspected injury. Based on this flexion view, anterior subluxation of C5 was clearly observed, confirming the diagnosis of a DF injury (Figure 4). Immediate cessation of flexion led to rapid improvement of his upper limb symptoms. Although surgery was planned, the patient requested to be transferred to his local hospital for continued care. He was transferred while wearing a cervical collar.

## Discussion

In this case, careful recognition of subtle imaging findings allowed a safe and accurate diagnosis under the supervision of an orthopaedic surgeon, highlighting key aspects in the detection and management of cervical DF injuries.

Two key clinical insights emerged from this case. (1) Subtle radiological findings - such as a facet tip fracture and minimal widening of the contralateral facet joint - can indicate underlying cervical instability. (2) The diagnostic process should not rely solely on initial imaging but must incorporate structured clinical protocols, including supervised dynamic testing when appropriate.

Although these findings may appear minor, they were key indicators of a DF injury in this case. According to the Allen-Ferguson classification, DF stage 2 injuries may present with "a small fleck of bone displaced from the posterior surface of the articular process" [1]. While cervical spine CT has become the standard for evaluating cervical trauma, several reports have described delayed dislocation and neurological symptoms, even in patients without detectable fractures on initial imaging [2,4-6]. These findings suggest that reliance on supine-position CT alone may be insufficient to rule out instability [3]. These seemingly minor findings highlight the need for heightened attention to subtle signs of instability in DF injuries.

Moreover, the diagnosis and management of cervical instability should not rely solely on initial imaging but must incorporate clinical evaluation and time-course observation. In patients with cervical spinal cord injury without radiographic abnormality, dynamic flexion-extension imaging has been recommended for assessing stability [8-10]. However, in the acute phase, pain and muscle spasm may limit the range of motion, and inappropriate testing may provoke or exacerbate neurological deficits [11,12]. In the present case, such imaging provoked transient neurological symptoms and revealed subluxation during flexion, which resolved in the neutral position. This strongly indicated instability. Even before encountering this case, our institution had already implemented a protocol to minimize diagnostic risk in suspected cervical spine injury.

This protocol consists of the following steps: (1) If the patient presents without spontaneous pain or neurological symptoms, active flexion and extension are first performed under supervision by a clinician. (2) If pain or neurological symptoms are provoked, dynamic flexion-extension radiography is performed under the direct supervision of an orthopaedic surgeon. (3) If no symptoms are elicited, dynamic imaging is conducted by a radiologic technologist. (4) At any point, if abnormal pain or neurological symptoms arise, the examination is stopped immediately, and further imaging is obtained. In this case, adherence to this protocol allowed for early recognition and timely treatment of cervical instability.

## Conclusions

In summary, this case demonstrates that, even subtle findings on supine-position CT - such as a facet tip fracture or slight widening of the contralateral facet joint - can provide critical clues to the presence of DF injury. Because spontaneous reduction may conceal instability, reliance on apparently stable CT findings alone risks delayed or missed diagnosis. In our patient, recognition of these subtle abnormalities prompted further evaluation, which confirmed instability before dislocation or neurological deterioration occurred. Importantly, the diagnostic process was supported by a structured protocol in which dynamic flexion-extension imaging was performed stepwise and only under direct physician supervision when symptoms arose. This careful approach ensured diagnostic accuracy while minimizing patient risk. This report highlights the value of combining meticulous interpretation of CT images with a cautious, supervised method of dynamic assessment, thereby enabling early recognition of cervical instability and improving patient safety in the evaluation of cervical spine trauma.
